# Computational Design of High-Performance U-Shaped Seismic Dampers Using Statistical Optimization

**DOI:** 10.3390/ma18235403

**Published:** 2025-11-30

**Authors:** Ignacio Ríos, Álvaro Gómez, Felipe Romero, Alexis Salas, Angelo Oñate, Carlos Lanziotti, Sebastián Andrés Toro, Laurent Duchêne, Víctor Tuninetti

**Affiliations:** 1 Department of Mechanical Engineering, Universidad de La Frontera, Temuco 4811230, Chile; ignacio.rios@ufrontera.cl (I.R.); a.gomez07@ufromail.cl (Á.G.); 2 Master Program in Engineering Sciences, Faculty of Engineering, Universidad de La Frontera, Temuco 4811230, Chile; 3 Departamento de Ingeniería Mecánica (DIM), Facultad de Ingeniería (FI), Universidad de Concepción, Concepción 4070409, Chile; fromero2018@udec.cl (F.R.); alesalas@udec.cl (A.S.); cmedinam@udec.cl (C.L.); 4 Department of Materials Engineering (DIMAT), Faculty of Engineering, Universidad de Concepción, Concepción 4070415, Chile; aonates@udec.cl; 5 Departamento de Ingeniería Mecánica, Universidad de Santiago de Chile, USACH, Av. Bernardo O’Higgins 3363, Santiago 9170016, Chile; sebastian.toroc@usach.cl; 6 Department ArGEnCo-MSM, University of Liège, 4000 Liège, Belgium; l.duchene@uliege.be

**Keywords:** seismic damper, U-shaped damper (USSD), hysteretic damper, energy dissipation, statistical optimization, Taguchi method, finite element analysis (FEA), structural engineering, seismic hazard mitigation, steel dampers

## Abstract

**Highlights:**

**What are the main findings?**

- ANOVA identifies height, thickness, and length as key damper design parameters.
- Optimized UD-M4 model yields 7-fold energy dissipation and 9-fold stiffness in-crease.

**What are the implications of the main findings?**

- Validated workflow replaces trial-and-error for next-gen seismic device design.
- New prototypes offer enhanced resilience and 50% better deformability.

**Abstract:**

Passive metallic dampers are critical for the seismic resilience of structures, yet their design has historically relied on incremental modifications rather than systematic optimization. This study introduces and validates a data-driven workflow that combines the Taguchi method with nonlinear finite element analysis to design novel U-shaped seismic dampers (USSDs) with superior performance. Building on an experimentally validated computational model from prior work, an L25 orthogonal array was employed to systematically investigate key geometric parameters, with an Analysis of Variance (ANOVA) identifying height, thickness, and length as the most influential factors on damper behavior. This statistical insight guided the creation of two optimized models, with the UD-M4 model demonstrating a nearly seven-fold increase in total energy dissipation (340.6 kJ vs. 51.2 kJ), a nine-fold increase in stiffness, and a 50% improvement in deformability compared to the commercial UD-40 baseline. The primary contribution of this work is the validation of an efficient statistical–computational methodology for the performance-based design of next-generation seismic protection devices, moving beyond traditional trial-and-error approaches.

## 1. Introduction

Large-scale seismic events pose a significant and ongoing threat to global infrastructure, frequently causing catastrophic financial and human losses, as exemplified by the major earthquakes in Chile [1,2] and Japan [3]. To mitigate these risks, the field of structural engineering has developed a variety of seismic protection devices, which can be broadly categorized as passive, active, semi-active, and hybrid systems [4]. Among these, passive energy dissipation systems have emerged as a particularly cost-effective, reliable, and maintenance-free solution for preserving structural integrity under dynamic loads [5]. The principles of energy dissipation are widely applied across many engineering fields to control unwanted vibrations [6], and in this context, these systems function by absorbing and dissipating seismic energy through various mechanisms, thereby reducing the dynamic response of the structure to which they are attached. Semi-active systems, such as magnetorheological dampers, offer a balance between the reliability of passive devices and the adaptability of active systems by enabling real-time adjustment of their rheological properties, providing adaptive energy dissipation under varying seismic demands [7,8].

Within the family of passive devices, metallic dampers that dissipate energy through the hysteretic behavior of materials have been widely adopted due to their robust performance and simplicity [9]. A wide array of geometries have been proposed and studied, with the specific geometry dictating the mechanism of energy dissipation. These include steel plates [10], vertical bars [11], and arch-shaped dampers [12]. Specific examples that have been widely implemented include Buckling-Restrained Braces (BRBs) designed to yield symmetrically in both tension and compression [13], Added Damping and Stiffness (ADAS) devices that utilize the flexural yielding of X-shaped plates [14], and Steel Slit Dampers (SSDs), which dissipate energy through shear and flexural deformation [15].

The U-shaped seismic damper (USSD), first proposed by Kelly, Skinner, and Heine [16], is a particularly effective type of metallic damper due to its inherent bidirectional energy dissipation capabilities. Subsequent research has primarily focused on incremental geometric modifications. For instance, Ebadi Jamkhaneh et al. [17] proposed a modification with symmetrically closed ends, demonstrating through experimental and numerical work that the damper’s thickness is a key geometric characteristic influencing performance. Qiu et al. [18] introduced an adaptation incorporating three perpendicular ribs of varying sizes, which increased the stiffness and strength of the device.

More recently, several innovative damping configurations have emphasized hybrid energy dissipation and self-centering behavior. Qu et al. [19] developed a dual self-centering friction damper combining coil springs, shape memory alloys, and a friction interface, demonstrating that recentering capacity and energy dissipation can be explicitly balanced. Liang et al. [20] introduced a parallel scheme incorporating both friction and viscous dampers to control cumulative and peak displacements in long-span suspension bridges. Naeem et al. [21] proposed a self-centering disc-slit damper integrating pre-compressed Belleville disc springs with slit-type metallic cores to reduce residual displacements. Naghshineh et al. [22] evaluated inline seismic friction dampers in reinforced concrete buildings, identifying key design factors influencing overstrength, ductility, and response modification capacity. Aldea et al. [23] examined skewed highway bridges equipped with hysteretic dampers using three-dimensional nonlinear finite element analysis and multiple-stripe fragility assessment. Farajiani et al. [24] demonstrated the structural resilience benefits of U-shaped metallic dampers through Endurance-Time Analysis, reinforcing their relevance to seismic performance enhancement.

Despite these developments toward systematically tuned hysteretic and recentering behavior, studies focusing on incremental geometric or conceptual enhancements generally do not employ systematic optimization strategies to guide shape selection or quantify the influence of geometric parameters. In parallel, other fields of vibration control have advanced optimization frameworks. For example, in vehicle dynamics, Frequency-Dependent Equivalent Impedance (FDEI) analysis has been used to guide the structural optimization of inertial suspension systems [25]. This contrast highlights the need for similarly analytics-driven methodologies in the optimization of hysteretic seismic dampers

Computational modeling via the finite element method (FEM) remains essential for capturing the nonlinear hysteretic response of metallic dampers. Recent work by Tuninetti et al. [1] presented a validated computational framework for modeling U-shaped dampers under cyclic loading, incorporating geometric nonlinearities and accurately predicting local stress, strain, and energy dissipation against experimental data. However, despite these advances, a critical review of the literature reveals two persistent limitations: (1) simplified geometric optimization approaches often fail to capture localized strain concentrations [26], and (2) insufficient meshing strategies remain common in finite element modeling, limiting strain accuracy [17,27]. These limitations underscore the absence of a systematic optimization methodology grounded in a rigorously validated numerical model.

This study addresses these gaps by presenting a workflow that integrates nonlinear finite element analysis with the Taguchi statistical method [28,29] to design and optimize novel USSDs. Leveraging the experimentally validated FEM from Tuninetti et al. [1], a Taguchi L25 orthogonal array is used to explore the design space, and an Analysis of Variance (ANOVA) identifies the most influential geometric factors. The optimized models, UD-M2 and UD-M4, demonstrate substantial improvements in energy dissipation capacity and deformability when compared to a widely used commercial baseline.

## 2. Methodology

The methodology for designing and optimizing the novel seismic dampers involved a multi-step, computationally driven process. This section provides a detailed account of the geometric prototyping, material characterization, finite element model setup, and the statistical framework used for the design and analysis of experiments.

### 2.1. Geometric Prototyping and Baseline Model

**Figure 1:**
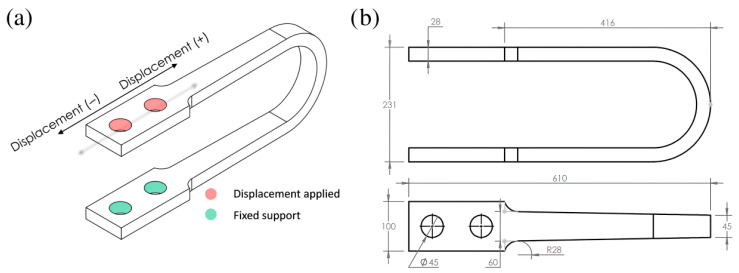
(**a**) UD-40 model with boundary conditions applied. (**b**) Upper and front views of UD-40 USSD (all dimensions in mm). Note: The internal lines indicate geometric transitions only; the damper is monolithic and fabricated from JIS SM490 structural steel. Reproduced from Tuninetti et al. [1], Applied Sciences, 14(22), 10238 (https://doi.org/10.3390/app142210238).

The baseline for this study is the commercial Nippon Steel UD-40 USSD (Figure 1). The initial geometric model was created in SolidWorks Student Edition based on technical drawings and dimensions reported in the literature. As represented in Figure 1b, the UD-40 model has a total length of 610 mm, a height of 231 mm, a thickness of 28 mm, and a primary width of 60 mm. The U-shaped geometry was selected because it represents a widely adopted industrial standard known for its stable hysteretic behavior and capacity to dissipate energy through rolling bending deformation, which minimizes the stress concentrations often found in sharp-cornered devices. The specific UD-40 model was chosen as the baseline for two primary reasons: first, it is the most extensively analyzed model in the existing literature, providing a wealth of comparative data for validation; second, as the smallest model in its commercial series, it represents a conservative baseline. Demonstrating significant performance improvements over this model provides a robust proof-of-concept for the optimization methodology.

### 2.2. Material Characterization and Constitutive Model

The damper material is JIS SM490 structural steel, with mechanical properties obtained from experimental tensile tests reported by Liu et al. [30]. To accurately capture the material’s behavior, the engineering stress–strain curve was converted to a true stress–strain curve. Subsequently, a bilinear kinematic hardening model was implemented. As detailed and experimentally validated in Tuninetti et al. [1], this model accurately reproduces the hysteretic response, with a Yield Strength of 403.3 MPa, a Young’s Modulus of 215.8 GPa, and a Tangent Modulus of 1324.7 MPa.

### 2.3. Finite Element Model Setup

The performance of each geometric model was evaluated via nonlinear finite element analysis (FEA), conducted using ANSYS Mechanical within Workbench environment (Static Structural module). To ensure the accurate capture of geometric nonlinearities inherent in large deformations, the large deflection setting was activated for all simulations under quasi-static cyclic loading.

**Figure 2:**
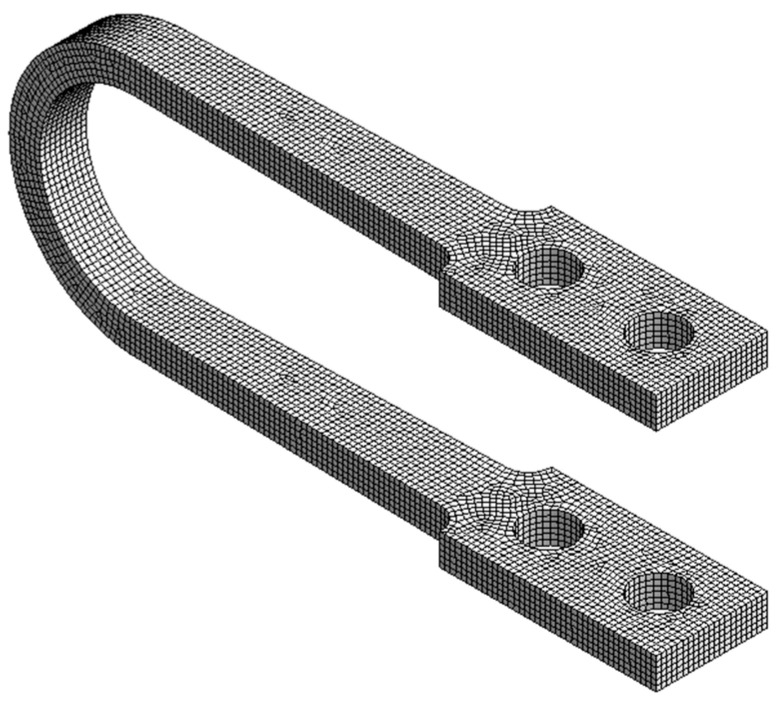
Finite element mesh of the UD-40 baseline damper model.

A hex-dominant meshing strategy was employed (Figure 2), as hexahedral elements provide more accurate results with fewer elements compared to tetrahedrons, especially in complex geometries. As detailed in our prior work [1], a rigorous mesh convergence analysis [31] using an adapted Richardson extrapolation method determined that a mesh with six elements through the damper’s thickness was optimal. Consequently, a high mesh density was strictly maintained to prevent the locking phenomena common in coarse meshes during bending and to ensure that the peak plastic strains at the curved sections were captured independent of mesh size. The final mesh for the baseline UD-40 contained 23,906 elements and was of high quality, with 87% of elements being hexahedral.

The boundary conditions were designed to simulate the damper’s installation in a structure, specifically assuming a standard inverted-V (chevron) bracing configuration. In this setup, the damper is rigidly connected between the apex of the steel braces and the floor beam above, such that inter-story drift imposes a purely longitudinal shear deformation on the device while the bracing elements restrict rotation. Accordingly, an embedded fixed support was applied to the inner surfaces of the lower bores, while a Remote Displacement boundary condition was applied to the inner surfaces of the upper bores. This condition enforced a guided longitudinal displacement along the x-axis while constraining rotation and vertical movement, simulating the rigid connection to the structural frame (see Figure 1a above). A quasi-static, 20-step cyclic loading protocol was applied, consisting of 10 cycles of progressively increasing displacement from ±10 mm to a maximum of ±100 mm. While the device is experimentally capable of 500 mm displacement, the ±100 mm range was selected as it corresponds to an inter-story drift of approximately 3.3% (for a 3 m story height). This value aligns with the Collapse Prevention limit state defined in seismic design codes such as ASCE 7-16 [32], making it the critical functional domain for optimization.

**Figure 3:**
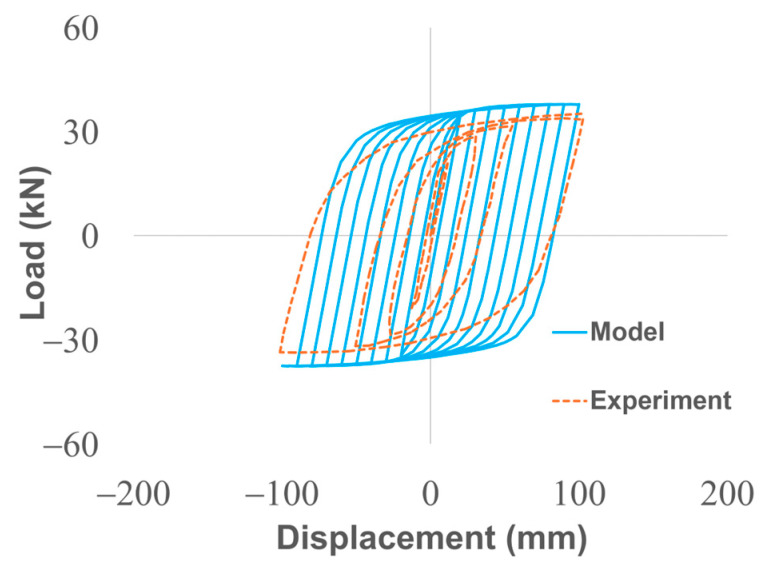
Validation of the computational model: Hysteresis curve comparing Model (solid blue) and Experimental (dashed orange) data (reproduced from Tuninetti et al. [1], Applied Sciences, 14(22), 10238; https://doi.org/10.3390/app142210238).

To ensure model reliability, the FEM was experimentally validated against cyclic loading tests on the UD-40 damper, as reported in Tuninetti et al. [1]. The validation compared simulated hysteresis curves (reaction force vs. displacement) and dissipated plastic strain energy against experimental data from Suzuki et al. [33], showing good agreement with an average relative error of 7.8% in reaction forces (Figure 3). The bilinear model slightly overpredicts strain hardening in early cycles due to its simplification of the true stress–strain curve, but this conservative error is acceptable for relative design comparisons and preliminary optimization.

A Taguchi L25 orthogonal array was selected for the Design of Experiments (DOE) to efficiently analyze the influence of four key geometric factors on damper performance. Each factor was assigned five distinct levels, as detailed in Table 1, resulting in 25 unique damper prototypes for simulation. The performance of each prototype was quantified using two response variables: Accumulated Equivalent Plastic Strain (AEPS) to measure deformability and Maximum Energy per Unit Volume to assess dissipation efficiency. Additionally, maximum stress and stiffness (Beer et al. [34]) are also computed for analysis.

The simulation results were statistically analyzed in Minitab 21 software (Minitab, LLC, State College, PA, USA). Data integrity was first confirmed by conducting a Ryan–Joiner test for normality and a Grubbs test to identify and remove outliers. Subsequently, an Analysis of Variance (ANOVA) was performed to determine which geometric factors had a statistically significant effect (defined by a *p*-value < 0.05) on the selected response variables.

#### Governing Equations and Performance Metrics

The numerical analysis solves the static equilibrium equation for the damper body Ω, neglecting inertial forces due to the quasi-static nature of the loading (Equation (1)).

(1) ∇⋅σ+b=0

where **σ** is the Cauchy stress tensor and ***b*** represents body forces. To model the onset of plastic deformation in the steel, the von Mises yield criterion is employed (Equation (2)).

(2) $$\sigma_{eq}=\sqrt{\frac{1}{2}\left[{\left(\sigma_{1}-\sigma_{2}\right)}^{2}+{\left(\sigma_{2}-\sigma_{3}\right)}^{2}+{\left(\sigma_{3}-\sigma_{1}\right)}^{2}\right]}\geq \sigma_{y}$$

where $\sigma_{eq}$ is the equivalent stress, $\sigma_{1,2,3}$ are the principal stresses, and $\sigma_{y}$ is the yield strength of the JIS SM490 steel (403.3 MPa).

The performance metrics reported in the Results were calculated directly from the hysteresis data. The initial elastic stiffness ($K_{e}$) was computed as the secant slope from the origin to the yield point:

(3) $$K_{e}=\frac{F_{y}}{\delta_{y}}$$

where $F_{y}$ and $\delta_{y}$ are the reaction force and displacement at the onset of yielding, respectively. The total dissipated energy ($E_{D}$) was calculated by numerically integrating the area enclosed by the force-displacement hysteresis loops:

(4) $$E_{D}=\int F\left(x\right)\;dx$$

## 3. Results

The results of the statistical optimization step and the subsequent comparative performance analysis are presented below. The findings are based on the computational simulation of 25 unique damper prototypes derived from the Taguchi L25 orthogonal array.

### 3.1. Identification of Key Design Factors via ANOVA

An Analysis of Variance (ANOVA) was conducted on the simulation results to quantitatively determine the influence of each geometric factor on the selected performance metrics. The normality of the response data was first confirmed using the Ryan–Joiner test (*p* > 0.100), and a single outlier was removed prior to analysis.

The ANOVA revealed that for Maximum Energy per Unit Volume, the damper’s height, thickness, and total length were all identified as statistically significant factors (*p*-value < 0.001), indicating a strong correlation with energy dissipation efficiency. For Accumulated Equivalent Plastic Strain (AEPS), the damper’s height was the most influential factor, with the analysis showing particular significance at its lowest level of 231 mm. In contrast, and as expected for the unidirectional loads investigated, the damper’s width was found to be statistically insignificant

### 3.2. Design of Optimized Damper Models

The statistical insights from the ANOVA guided the formulation of two new damper designs. The first model, UD-M2, was designed by combining the optimal levels of the statistically significant geometric parameters identified in the analysis. The second model, UD-M4, was selected directly from the L25 array results, as it was identified via a Grubbs test as a high-performance statistical outlier. While the UD-M2 represents a design optimized by combining the best-performing main-effect levels, the UD-M4 represents a specific combination from the L25 array (Height = 297, Thickness = 55) that yielded exceptional energy dissipation. Its selection highlights that potential interaction effects, not captured by the main effects analysis, can lead to highly effective, non-obvious design solutions.

**Figure 4:**
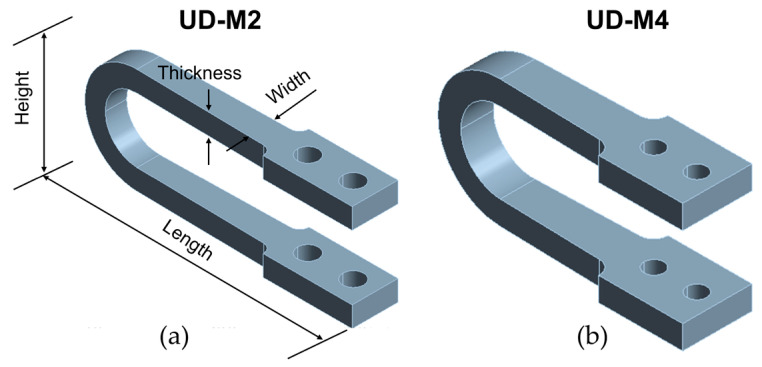
Three-dimensional models of the optimized U-shaped seismic dampers: (**a**) UD-M2 and (**b**) UD-M4.

The final dimensions of both optimized models are detailed in Table 2 and visualized in Figure 4.

To quantify the performance improvements, the optimized UD-M2 and UD-M4 models were subjected to the same computational analysis as the UD-40 baseline. A comprehensive summary of the key performance metrics comparing the three dampers is presented in Table 3.

**Figure 5:**
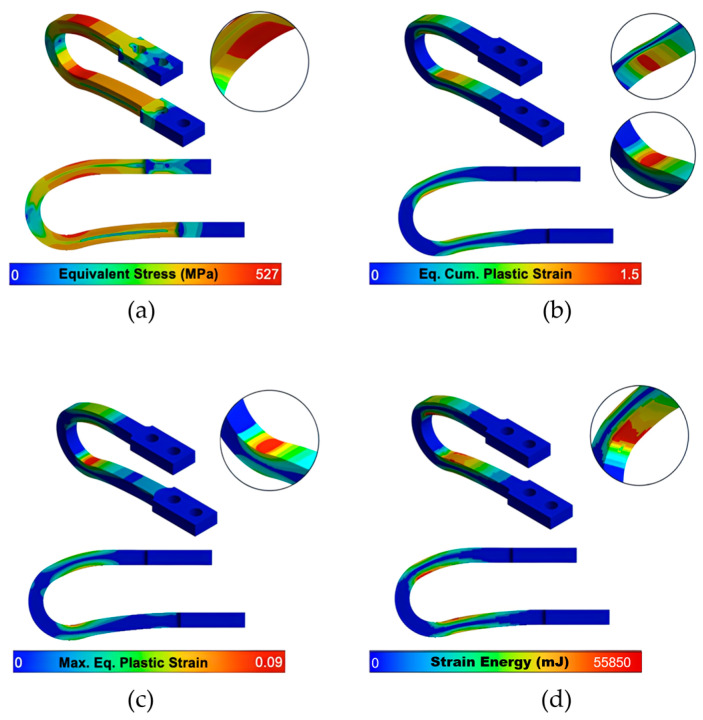
UD-M2’s contours of (**a**) Maximum equivalent stress, (**b**) Equivalent (Eq.) Cumulative (Cum.) plastic strain (AEPS, unitless), (**c**) Maximum (Max.) Equivalent plastic strain (unitless) and (**d**) Strain energy after 10 cycles of increasing displacement.

**Figure 6:**
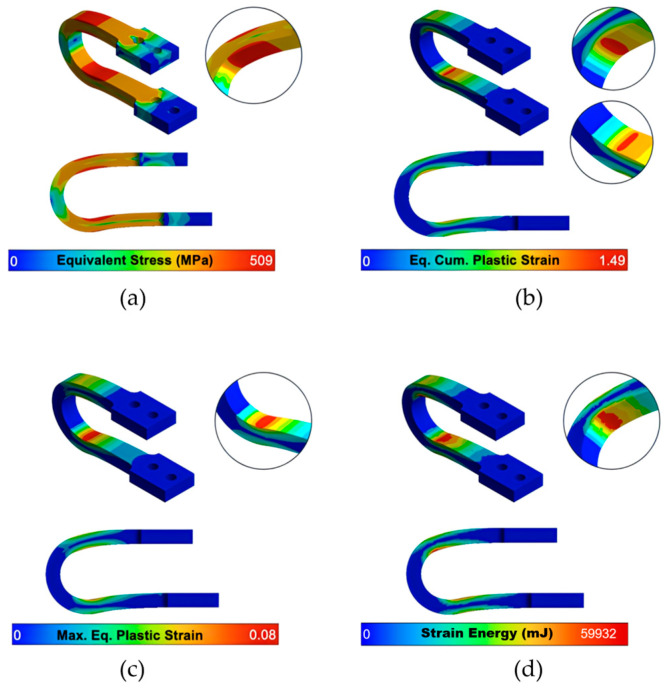
UD-M4’s contours of (**a**) Maximum equivalent stress, (**b**) Equivalent Cumulative plastic strain (AEPS, unitless), (**c**) Maximum equivalent plastic strain (unitless) and (**d**) Strain energy after 10 cycles of increasing displacement.

A detailed analysis of the local stress and strain fields at maximum displacement is provided in the contour plots in Figure 5 and Figure 6. While the optimized UD-M2 and UD-M4 models exhibit higher peak equivalent stresses (526.6 MPa and 509.3 MPa, respectively) compared to the 475.2 MPa of the baseline, their stress distribution is similar. This confirms that the optimization did not introduce unintended stress concentrations in new locations. Importantly, all peak stresses remain safely below the material’s ultimate tensile strength (641.4 MPa).

Crucially, while all models concentrate plastic strain in the intended yielding zones, the optimized dampers demonstrate vastly improved deformability. The UD-M2 and UD-M4 models achieve a maximum Accumulated Equivalent Plastic Strain (AEPS) of 1.50 and 1.49, respectively—a nearly 50% increase over the 0.925 value of the UD-40 baseline.

**Figure 7:**
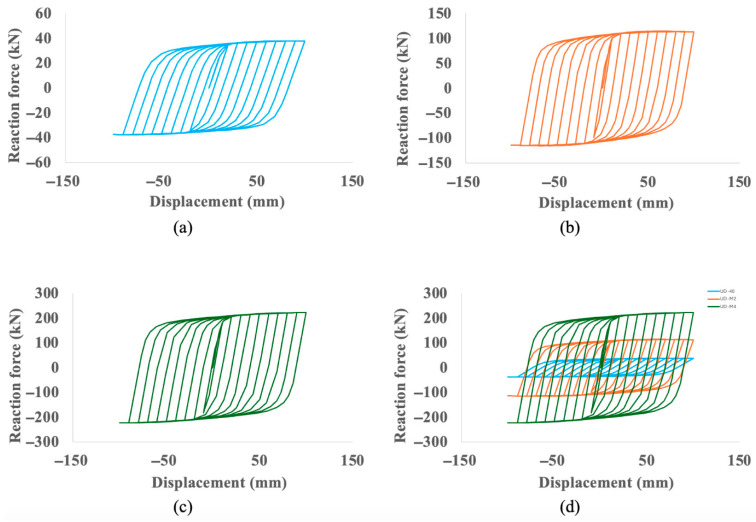
Hysteresis curve for (**a**) UD-40 (reproduced from Tuninetti et al. [1], Applied Sciences, 14(22), 10238; https://doi.org/10.3390/app142210238), (**b**) UD-M2, (**c**) UD-M4 and (**d**) comparison between the three curves.

The most significant performance gains are demonstrated in the hysteretic behavior of the dampers, as shown in Figure 7. The area enclosed by the force-displacement loops, which represents the energy dissipated per cycle, is substantially larger for the optimized models as a direct result of their increased strength and stiffness. The baseline UD-40 reaches a peak reaction force of approximately 38 kN, whereas the UD-M2 and UD-M4 models reach peak forces of approximately 110 kN and over 200 kN, respectively, at the same maximum displacement. The comparative plot (Figure 7d) visually confirms the dramatic increase in stiffness, with the UD-M4 exhibiting a much steeper elastic slope.

**Figure 8:**
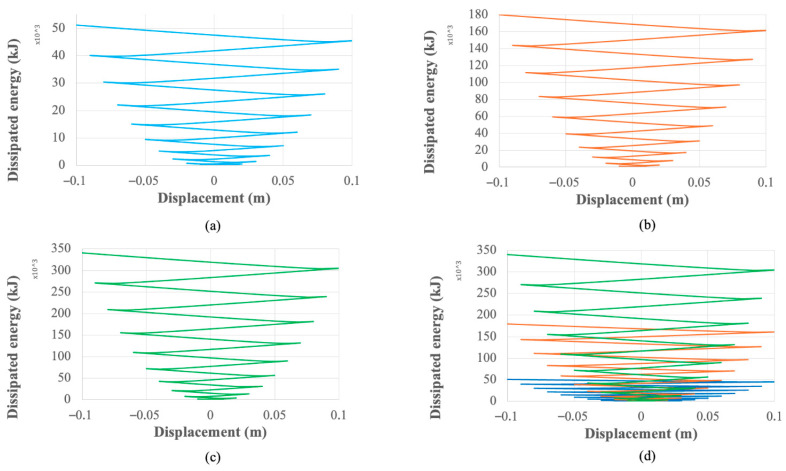
Cumulative energy dissipation for (**a**) UD-40 (reproduced from Tuninetti et al. [1], Applied Sciences, 14(22), 10238; https://doi.org/10.3390/app142210238), (**b**) UD-M2, (**c**) UD-M4 and (**d**) comparison between the dampers.

**Figure 9:**
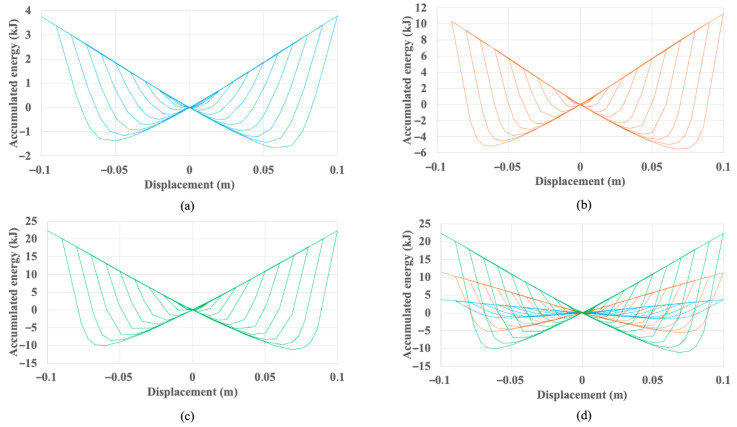
Work vs. displacement of (**a**) UD-40, (**b**) UD-M2, (**c**) UD-M4 and (**d**) comparison between the three dampers.

This enhanced hysteretic response translates directly into superior cumulative energy dissipation, which is quantified in Figure 8. Over the 10-cycle protocol, the baseline UD-40 dissipates a total of 51.2 kJ. In contrast, the UD-M2 dissipates 179.8 kJ (a 3.5-fold increase), and the UD-M4 dissipates 340.6 kJ (a nearly seven-fold increase). The accumulated work plots in Figure 9 further illustrate this enhanced capacity on a per-cycle basis, showing the UD-40 reaches a peak of approximately 4 kJ per cycle, while the UD-M4 exceeds 20 kJ.

## 4. Discussion

This study successfully demonstrated that a statistical optimization workflow can produce novel U-shaped seismic dampers with performance characteristics vastly superior to existing commercial designs. The following sections interpret these performance gains, place the methodology in the context of existing research, and discuss the practical implications of the findings.

### 4.1. Interpretation of Performance Gains

The results clearly validate the statistical workflow as a powerful tool for navigating complex design trade-offs. The optimization did not merely improve one performance metric but successfully decoupled and enhanced three competing characteristics: stiffness, energy dissipation, and deformability.

The massive increase in stiffness and strength is the most direct outcome, as evidenced by the hysteretic behavior (Figure 7). The UD-M4 model’s ability to generate over five times the peak reaction force of the baseline (over 200 kN vs. 38 kN) is a direct consequence of its optimized geometry, particularly its increased thickness, aligning with the mechanical principle that bending stiffness is proportional to thickness cubed. This is quantified by the UD-M4’s nine-fold increase in calculated stiffness (21,163 N/mm vs. 2311 N/mm). This enhanced stiffness directly translates into a far superior capacity for energy dissipation, with the UD-M4 achieving a nearly seven-fold increase in total dissipated energy (Figure 8).

Crucially, these gains were not achieved at the expense of ductility. A common engineering challenge is that increased stiffness often leads to reduced deformability. However, our most significant finding is that the optimization workflow simultaneously increased deformability by approximately 50%, as measured by the Accumulated Equivalent Plastic Strain (AEPS). The contour plots (Figure 5 and Figure 6) confirm that despite much higher internal stresses, the plastic strain remains concentrated in the intended yielding zones. This dual improvement results in dampers that are not only stronger and more effective but also more resilient and capable of sustaining greater cumulative plastic damage.

Furthermore, based on the initial elastic stiffness (Ke) derived from the hysteresis curves (Figure 7) and the mass of the devices, the fundamental natural frequency of the baseline component is estimated to be approximately 60 Hz. This value is well above the typical frequency range of seismic ground motions (0–10 Hz) [35], ensuring that during low-amplitude events where the damper remains elastic, there is no risk of resonance with the excitation frequency.

### 4.2. Comparison with Existing Research and Methodological Considerations

Our foundational work established the need for a fine mesh with six elements through the thickness for high-accuracy results. This finding is critically supported when compared to other studies. For instance, Atasever et al. [36] used four elements and reported errors close to 10% in equivalent plastic strain, while [17] used only one element in the thickness and reported a 14% higher damper stiffness and 9.4% lower dissipated energy compared to experimental data, a discrepancy they attribute to mesh size. This comparison validates our more rigorous meshing strategy as essential for the reliable predictions needed for optimization. The experimental validation in Tuninetti et al. [1] further distinguishes this work, providing a robust basis for the statistical optimization here, unlike prior simplified models lacking such verification (e.g., Khatibinia et al. [26]). The novelty lies in integrating this validated FEM with Taguchi-ANOVA to systematically identify and exploit geometric influences, yielding non-incremental designs with decoupled performance improvements—advancing beyond trial-and-error or hybrid concepts (e.g., Qu et al., [19]).

The use of a bilinear kinematic hardening model, while computationally efficient, represents a known simplification of the true stress–strain curve. As detailed in our foundational work, this model tends to overestimate strain hardening in the initial yielding phase, resulting in a slight overprediction of the reaction forces with an average relative error of 7.8% when compared to experimental data.

For the purposes of design and selection of hysteretic dampers, this level of error is considered acceptable for several key reasons. First, the model’s primary function in this study was to compare the relative performance of 25 different designs. Since the systematic overprediction of stiffness is applied consistently across all simulations, the resulting performance ranking and the identification of the superior models (UD-M2, UD-M4) remain valid. Second, in the context of preliminary engineering design, computational models are intended to guide engineers toward promising solutions that are later validated experimentally; an error of less than 10% is well within the typical bounds for such analyses. Finally, a slight overestimation of the damper’s stiffness is a conservative error, ensuring that the real-world device is unlikely to be overloaded based on these predictions.

### 4.3. Practical Implications and Future Work

From a practical standpoint, the new models offer a clear engineering choice: the UD-M2 provides a more material-efficient design with still-dramatic performance gains, while the UD-M4 offers maximum performance for critical applications. The selection would depend on the specific demands of the structure and economic constraints.

While the computational findings are considered highly reliable due to the validated modeling approach, the essential next step for facilitating real-world adoption is the experimental validation of the UD-M2 and UD-M4 prototypes. Following this validation, future work must focus on specific engineering applications. The high stiffness and dissipation capacity of the UD-M4, for instance, make it an ideal candidate for seismic retrofitting of vulnerable structures, such as non-ductile concrete frames, unreinforced masonry (URM) buildings, or structures with soft-story configurations. However, given the high stiffness of the UD-M4 model relative to masonry, its application in URM retrofits would strictly require a steel sub-frame or reinforced concrete collector system. This is necessary to distribute the high reaction forces and prevent localized crushing failure of the brickwork at the connection points. Furthermore, the device’s high stiffness makes it well-suited for critical infrastructures like hospitals, data centers, and key transportation bridges, where limiting inter-story drift is paramount.

To facilitate this adoption by practicing engineers, key implementation challenges must be addressed. The massive increase in reaction forces (over 210 kN for the UD-M4) necessitates a dedicated methodological study on the design of high-capacity connection systems (e.g., gusset plates and bolt configurations) to ensure loads are transferred without premature failure. Concurrently, cost–benefit analyses, investigations into manufacturing challenges, and the development of simplified, performance-based design guidelines will be required.

Additionally, while this study optimized the damper under pure in-plane longitudinal loading, real-world installation imperfections may introduce accidental eccentricity. Therefore, future experimental validation should consider out-of-plane and torsional loading scenarios to assess the damper’s stability under complex multi-axial seismic demands. Future research should also focus on the integration of these optimized dampers into structural systems, such as in chevron bracing configurations or as part of replaceable coupling beams, to validate their system-level performance. Furthermore, the current optimization was conducted under quasi-static loading conditions. For applications involving high-velocity impact or shock loads, the strain-rate sensitivity of the steel (viscoplasticity) becomes significant. Future research should therefore incorporate rate-dependent material models to validate the damper’s performance under dynamic impact scenarios.

Lastly, future work could address the inherent limitations of this damper type. Since they lack re-centering capability, innovations could explore hybrid designs that combine the high energy dissipation of these optimized dampers with the re-centering properties of mechanisms like shape memory alloys or pre-tensioned tendons [37,38]. Regarding system-level dynamics, it is noted that while this study focused on component-level hysteretic behavior, future research will incorporate these optimized dampers into a full structural model to perform a modal analysis. This next step is essential to quantify the dampers’ influence on the natural periods and damping ratios of multi-story frames under dynamic excitation.

## 5. Conclusions

This research successfully developed and validated a statistical–computational workflow as a powerful tool for designing superior U-shaped seismic dampers, moving the field beyond traditional incremental modifications. By building on an experimentally verified FEM (Tuninetti et al. [1]), this study demonstrated that a systematic, data-driven approach can yield new designs with performance characteristics that significantly surpass existing commercial models. The optimized UD-M4 model, for instance, exhibited a nine-fold increase in stiffness (from 2311 N/mm to 21,163 N/mm) and a seven-fold increase in total energy dissipation (from 51.2 kJ to 340.6 kJ) over the baseline. Crucially, these substantial gains were achieved while simultaneously increasing the damper’s deformability by approximately 50%.

The primary contributions of this work are twofold: First, the workflow produced two distinct high-performance damper designs: the balanced UD-M2 model and the maximum-performance UD-M4 model. Second, it quantitatively identified height, thickness, and length as the key geometric drivers of USSD performance under unidirectional loads, providing clear design guidance for future iterations. Together, these findings offer a validated computational blueprint for next-generation dampers, positioning the UD-M2 and UD-M4 prototypes as promising candidates for future seismically resilient structures.

## Figures and Tables

**Table 1:** Factors and Levels for the Taguchi L25 Orthogonal Array.

Variables	Height (mm)	Width (mm)	Thickness (mm)	Total Length (mm)
Level 1 (UD40)	231	60	28	610
Level 2 (UD45)	297	77	36	785
Level 3 (UD50)	330	85	40	872
Level 4 (UD55)	371	96	45	981
Level 5 (UD60)	453	118	55	1199

**Table 2:** Dimensions of the new optimized damper models, derived from the ANOVA results.

Model	Height (mm)	Width (mm)	Thickness (mm)	Total Length (mm)
UD-M2	231	60	45	610
UD-M4	297	96	55	610

**Table 3:** Comparative performance results for the baseline (UD-40) and optimized (UD-M2, UD-M4) dampers.

Model	Maximum. Equivalent Stress (MPa)	AEPS	Peak Reaction Force (kN)	Maximum Dissipated Energy (kJ)	Secant Stiffness (N/mm)
UD-40	475.2	0.925	38	51.2	2311
UD-M2	526.6	1.500	110	179.8	11,908
UD-M4	509.3	1.490	210	340.6	21,163

## Data Availability

The original contributions presented in this study are included in the article. Further inquiries can be directed to the corresponding author.
